# Oral microbiome diversity in chimpanzees from Gombe National Park

**DOI:** 10.1038/s41598-019-53802-1

**Published:** 2019-11-22

**Authors:** Andrew T. Ozga, Ian Gilby, Rebecca S. Nockerts, Michael L. Wilson, Anne Pusey, Anne C. Stone

**Affiliations:** 1 Center for Evolution and Medicine, Arizona State University, Tempe, Arizona USA; 2 Institute of Human Origins, Arizona State University, Tempe, Arizona USA; 3 Halmos College of Natural Sciences and Oceanography, Nova Southeastern University, Fort Lauderdale, Florida USA; 4 School of Human Evolution and Social Change, Arizona State University, Tempe, Arizona USA; 5 Department of Anthropology, University of Minnesota, Minneapolis, Minnesota USA; 6 Department of Ecology, Evolution, and Behavior, University of Minnesota, Minneapolis, Minnesota USA; 7 Department of Evolutionary Anthropology, Duke University, Durham, North Carolina USA

**Keywords:** Anthropology, Microbiome

## Abstract

Historic calcified dental plaque (dental calculus) can provide a unique perspective into the health status of past human populations but currently no studies have focused on the oral microbial ecosystem of other primates, including our closest relatives, within the hominids. Here we use ancient DNA extraction methods, shotgun library preparation, and next generation Illumina sequencing to examine oral microbiota from 19 dental calculus samples recovered from wild chimpanzees (*Pan troglodytes schweinfurthii*) who died in Gombe National Park, Tanzania. The resulting sequences were trimmed for quality, analyzed using MALT, MEGAN, and alignment scripts, and integrated with previously published dental calculus microbiome data. We report significant differences in oral microbiome phyla between chimpanzees and anatomically modern humans (AMH), with chimpanzees possessing a greater abundance of Bacteroidetes and Fusobacteria, and AMH showing higher Firmicutes and Proteobacteria. Our results suggest that by using an enterotype clustering method, results cluster largely based on host species. These clusters are driven by *Porphyromonas* and *Fusobacterium* genera in chimpanzees and *Haemophilus* and *Streptococcus* in AMH. Additionally, we compare a nearly complete *Porphyromonas gingivalis* genome to previously published genomes recovered from human gingiva to gain perspective on evolutionary relationships across host species. Finally, using shotgun sequence data we assessed indicators of diet from DNA in calculus and suggest exercising caution when making assertions related to host lifestyle. These results showcase core differences between host species and stress the importance of continued sequencing of nonhuman primate microbiomes in order to fully understand the complexity of their oral ecologies.

## Introduction

The human oral cavity contains an estimated 600 different microbial species^1^. The oral microbiome also exhibits strong interpersonal and population-specific variation across the globe^2,3^, while at the same time differentiating between healthy and diseased oral states^4^. Advances in next generation sequencing and bioinformatic analyses have allowed researchers to study the oral microbiota of modern as well as historic and prehistoric populations through the investigation of dental calculus (calcified plaque). Dental calculus is commonly found in living populations without adequate dental care as well as archaeological skeletal assemblages and has been estimated to contain 200 million cells per milligram^5,6^ consisting of host cells^7^, bacteria, viruses, and occasionally dietary information. This biological resource has been used to answer many biological and anthropological questions addressing such topics as Neanderthal diet and behavior^8,9^, the evolution of antibiotic resistance genes in oral pathogens^10^, and the bacterial composition of pre-contact Puerto Rican dental calculus^11^.

Although the oral microbiome has been shown to be associated with host health and disease^1^ and exhibit incredible diversity across the globe in humans^2,12–14^, little focus has been paid to nonhuman primate oral microbiomes. To date, Weyrich *et al*.^9^ is the only study to include a historic oral microbiome sample from *Pan troglodytes*. As for modern microbiomes, a single study examined modern ape oral ecosystems through saliva, which uncovered a greater similarity between baboon and chimpanzee species (Sierra Leone and Democratic Republic of Congo) when compared to human caretakers from each sanctuary facility^15^. This research further suggested that a captive environment drastically impacts the primate oral ecology^15^. Outside of the oral cavity, specifically within the primate gut, clusters known as ‘enterotypes’ show that regardless of geographic origin, gorillas and chimpanzees share a Prevotella-dominated gut signature with modern humans^16–18^. These clusters were generally thought to be associated with the long term dietary practices of the host^17^. However, the enterotype concept is somewhat controversial and a sole reliance on enterotype clustering classifications may obscure critical microbial variation^19^. The existence of these enterotype clusters within the human and chimpanzee oral cavity has yet to be explored.

In this study, we characterize the microbiota in the oral cavity of wild chimpanzees using next generation shotgun sequencing of dental calculus. We first focus on differences in abundance between anatomically modern humans (AMH) and chimpanzees at the phylum and genus levels as well as shared types between groups. Second, we address the question of whether chimpanzee oral microbiota adhere to an enterotyping pattern as seen within primate gut microbiomes. Third, we reconstruct a full *Porphyromonas gingivalis* genome from a single chimpanzee and compare it to previously published genomes. Lastly, since the chimpanzees at Gombe have been observed for more than fifty years and their diet is well documented^20,21^, we map sequence data indicative of diet to understand whether such methods are useful for inferring lifestyle. This research helps to situate the previously unexplored chimpanzee oral microbiota from dental calculus with other historic and prehistoric human samples in an effort to understand the complexity of microbial diversity across the primate oral ecosystem.

## Results

### Sequencing statistics and MetaPhlAn2 analyses

For initial analyses we examined data from 19 Gombe chimpanzee calculus samples and two sets of comparative data from a total of 46 individuals. The first set includes 25 historic AMH calculus samples^22^ and the second set has data from 21 samples including Neanderthals as well as prehistoric, historic, and contemporary AMH, and a nonhuman sample from a historic chimpanzee^9^ (Table 1). A total of 95% of raw sequence reads passed adapter trimming, merging, and QC > 20 for the data from Gombe chimpanzees reported here. For the previously published datasets, the percentages of reads passing the same quality control thresholds were slightly lower (93% in the AMH dental calculus samples from Mann *et al*.^22^, and 69% from the Neanderthal/AMH/chimpanzee samples from Weyrich *et al*.^9^).

**Table 1:** Sample details including geographic location, age, sequencing statistics and reads mapped using both MetaPhlAn2 and MALT.

Sample Name	Species	Detail	Site	Country	Estimated Age (approx)	Raw Paired Reads	Trimmed, Merged, Q20 Reads	Total Reads Mapped with MetaPhlAn2	Percent MetaPhlAn2	Total Reads Mapped with MALT/MEGAN	Percent MALT/MEGAN	Normalized Reads from MALT/MEGAN used for Abundance	Citation
AFR_HG_12014	*Homo Sapiens*	Hunter-Gatherers	Dudka	Poland	7550BP	249,435	194,548	846	0.435%	7,840	4.030%	Not Analyzed	Weyrich *et al*.^9^
AFR_HG_12017	*H*. *Sapiens*	Hunter-Gatherers	Dudka	Poland	7550BP	136,233	117,206	345	0.294%	4,050	3.455%	Not Analyzed	Weyrich *et al*.^9^
AFR_IR_13232	*H*. *Sapiens*	Industrial Revolution	Stuttgart-Mühlhausen I	Germany	1850CE	185,781	144,526	902	0.624%	9,867	6.827%	Not Analyzed	Weyrich *et al*.^9^
AFR_IR_13234	*H*. *Sapiens*	Industrial Revolution	Stuttgart-Mühlhausen I	Germany	1850CE	13,547,243	11,546,907	19,924	0.173%	553,985	4.798%	Not Analyzed	Weyrich *et al*.^9^
AFR_JEWB_8812	*H*. *Sapiens*	Historic	The Royal College of Surgeons, England	England	750CE	84,741	65,184	264	0.405%	3,481	5.340%	Not Analyzed	Weyrich *et al*.^9^
AFR_JEWB_8824	*H*. *Sapiens*	Historic	The Royal College of Surgeons, England	England	750CE	101,309	85,279	234	0.274%	4,517	5.297%	Not Analyzed	Weyrich *et al*.^9^
AFR_LBK_12824	*H*. *Sapiens*	Early Neolithic	Stuttgart-Mühlhausen I	Germany	7440BP	53,145	44,620	96	0.215%	1,891	4.238%	Not Analyzed	Weyrich *et al*.^9^
AFR_LBK_12826	*H*. *Sapiens*	Early Neolithic	Stuttgart-Mühlhausen I	Germany	7440BP	171,540	136,698	186	0.136%	4,072	2.979%	Not Analyzed	Weyrich *et al*.^9^
AFR_LBK_12829	*H*. *Sapiens*	Early Neolithic	Stuttgart-Mühlhausen I	Germany	7440BP	204,481	176,469	358	0.203%	8,962	5.079%	Not Analyzed	Weyrich *et al*.^9^
AFRICAN1	*H*. *Sapiens*	Neolithic	Cape Town vicinity	Sudan	5kBP	1,175,551	3,661	3	0.082%	1,006	27.479%	Not Analyzed	Weyrich *et al*.^9^
AFRICAN2	*H*. *Sapiens*	Neolithic	Cape Town vicinity	Sudan	5kBP	12,036,888	40,915	2	0.005%	1,145	2.798%	Not Analyzed	Weyrich *et al*.^9^
AFRICAN3	*H*. *Sapiens*	Pre-pastoralist	Cape Town vicinity	South Africa	1000BP	8,495,412	822,451	644	0.078%	26,887	3.269%	Not Analyzed	Weyrich *et al*.^9^
AFRICAN5	*H*. *Sapiens*	Hunter-Gatherers	Cape Town vicinity	South Africa	4–6kBP	18,909,969	3,024,439	715	0.024%	165,770	5.481%	Not Analyzed	Weyrich *et al*.^9^
AFRICAN6	*H*. *Sapiens*	Hunter-Gatherers	Cape Town vicinity	South Africa	4–6kBP	11,516,626	319,036	181	0.057%	7,454	2.336%	Not Analyzed	Weyrich *et al*.^9^
AFRICAN7	*H*. *Sapiens*	Pre-pastoralist	Cape Town vicinity	South Africa	1000BP	7,715,048	2,693,550	3,635	0.135%	95,219	3.535%	Not Analyzed	Weyrich *et al*.^9^
12873_Chimp	*Pan troglodytes verus*	Modern	Gala Forest	Sierra Leonne	<100BP	931,404	855,550	0	0.000%	855,550	100.000%	Not Analyzed	Weyrich *et al*.^9^
ELSIDRON1	*H*. *Neanderthalensis*	Paleolithic	El Sidron cave	Spain	49kBP	53,186,534	51,447,208	63,374	0.123%	1,488,051	2.892%	104,094	Weyrich *et al*.^9^
ELSIDRON2	*H*. *Neanderthalensis*	Paleolithic	El Sidron cave	Spain	49kBP	51,079,301	48,820,793	133,748	0.274%	1,926,473	3.946%	104,110	Weyrich *et al*.^9^
Modern C10	*H*. *Sapiens*	Modern	Adelaide	Australia	Modern	346,022	282,097	0	0.000%	282,097	100.000%	Not Analyzed	Weyrich *et al*.^9^
SPYNEW (Spy2)	*H*. *Neanderthalensis*	36k ybp	Spy Cave	Belgium	36kBP	6,126,530	3,899,961	6,966	0.179%	178,063	4.566%	104,140	Weyrich *et al*.^9^
SPYOLD (Spy1)	*H*. *Neanderthalensis*	36k ybp	Spy Cave	Belgium	36kBP	18,367,108	17,328,351	14,578	0.084%	1,322,737	7.633%	104,127	Weyrich *et al*.^9^
C214Calc	*H*. *Sapiens*	Chalcolithic Period	Camino del Molino	Spain	2340–2920BP	8,281,186	7,356,367	37,791	0.514%	298,123	4.053%	104,127	Mann *et al*.^22^
C53Calc	*H*. *Sapiens*	Chalcolithic Period	Camino del Molino	Spain	2340–2920BP	22,466,043	19,509,074	118,930	0.610%	719,222	3.687%	104,085	Mann *et al*.^22^
F1948Calc	*H*. *Sapiens*	Caribbean Late Ceramic	Anse a la Gourde	Guadeloupe	975–1375CE	9,297,892	8,559,763	62,555	0.731%	571,977	6.682%	104,104	Mann *et al*.^22^
F349ACalc	*H*. *Sapiens*	Caribbean Late Ceramic	Anse a la Gourde	Guadeloupe	975–1375CE	11,373,256	10,869,468	21,294	0.196%	355,407	3.270%	104,121	Mann *et al*.^22^
H10Calc	*H*. *Sapiens*	Bronze Age	Khövsgöl	Mongolia	2.7–3.5kBP	13,944,283	12,164,338	78,145	0.642%	548,149	4.506%	104,099	Mann *et al*.^22^
H24Calc	*H*. *Sapiens*	Bronze Age	Khövsgöl	Mongolia	2.7–3.5kBP	11,681,424	11,030,623	68,709	0.623%	505,737	4.585%	104,112	Mann *et al*.^22^
KT05Calc-PE	*H*. *Sapiens*	Multi-period	Kilteasheen	Ireland	1250CE	11,616,921	10,921,610	30,432	0.279%	442,345	4.050%	104,100	Mann *et al*.^22^
KT08Calc-PE	*H*. *Sapiens*	Multi-period	Kilteasheen	Ireland	1250CE	10,485,093	10,222,950	156,114	1.527%	989,322	9.677%	104,116	Mann *et al*.^22^
KT09Calc-PE	*H*. *Sapiens*	Multi-period	Kilteasheen	Ireland	1250CE	10,735,529	10,333,839	22,080	0.214%	355,899	3.444%	104,106	Mann *et al*.^22^
KT13Calc-PE	*H*. *Sapiens*	Multi-period	Kilteasheen	Ireland	1250CE	13,490,438	12,973,179	52,168	0.402%	477,881	3.684%	104,104	Mann *et al*.^22^
KT14Calc-PE	*H*. *Sapiens*	Multi-period	Kilteasheen	Ireland	1250CE	10,091,518	9,768,412	42,150	0.431%	381,478	3.905%	104,115	Mann *et al*.^22^
KT24Calc-PE	*H*. *Sapiens*	Multi-period	Kilteasheen	Ireland	1250CE	14,648,490	14,160,655	56,697	0.400%	520,966	3.679%	104,106	Mann *et al*.^22^
KT25Calc-PE	*H*. *Sapiens*	Multi-period	Kilteasheen	Ireland	1250CE	12,000,822	11,720,117	46,817	0.399%	492,351	4.201%	104,102	Mann *et al*.^22^
KT26Calc-PE	*H*. *Sapiens*	Multi-period	Kilteasheen	Ireland	1250CE	13,119,609	12,685,752	67,533	0.532%	628,550	4.955%	104,113	Mann *et al*.^22^
KT28Calc-PE	*H*. *Sapiens*	Multi-period	Kilteasheen	Ireland	1250CE	12,535,615	12,223,396	34,169	0.280%	444,944	3.640%	104,080	Mann *et al*.^22^
KT29Calc-PE	*H*. *Sapiens*	Multi-period	Kilteasheen	Ireland	1250CE	13,032,739	12,674,361	103,169	0.814%	592,379	4.674%	104,119	Mann *et al*.^22^
KT31Calc-PE	*H*. *Sapiens*	Multi-period	Kilteasheen	Ireland	1250CE	19,085,955	18,455,566	161,997	0.878%	1,219,599	6.608%	104,098	Mann *et al*.^22^
KT32Calc-PE	*H*. *Sapiens*	Multi-period	Kilteasheen	Ireland	1250CE	12,193,757	11,844,042	90,895	0.767%	605,303	5.111%	104,118	Mann *et al*.^22^
KT36Calc-PE	*H*. *Sapiens*	Multi-period	Kilteasheen	Ireland	1250CE	11,353,735	10,963,468	70,503	0.643%	565,652	5.159%	104,126	Mann *et al*.^22^
NF217Calc	*H*. *Sapiens*	Late Prehistoric	Norris Farms	United States	1300CE	5,780,869	5,102,963	25,960	0.509%	273,215	5.354%	104,135	Mann *et al*.^22^
NF47Calc	*H*. *Sapiens*	Late Prehistoric	Norris Farms	United States	1300CE	5,972,509	5,302,830	37,756	0.712%	250,231	4.719%	104,080	Mann *et al*.^22^
S108Calc	*H*. *Sapiens*	Historic	Middenbeemster	Netherlands	1850CE	6,961,981	5,776,749	20,011	0.346%	217,949	3.773%	104,039	Mann *et al*.^22^
S40Calc	*H*. *Sapiens*	Samdzong	Samdzong	Nepal	400–650CE	8,646,847	7,730,429	48,349	0.625%	471,685	6.102%	104,103	Mann *et al*.^22^
S41Calc	*H*. *Sapiens*	Samdzong	Samdzong	Nepal	400–650CE	9,599,653	7,672,495	70,618	0.920%	574,702	7.490%	104,115	Mann *et al*.^22^
S454Calc	*H*. *Sapiens*	Historic	Middenbeemster	Netherlands	1850CE	7,453,959	6,223,507	23,864	0.383%	334,235	5.371%	104,118	Mann *et al*.^22^
02C	*Pan troglodytes schweinfurthii*	Modern	Gombe	Tanzania	<100BP	10,359,380	9,962,780	61,926	0.622%	418,604	4.202%	104,142	Current Publication
04C	*P*. *t*. *schweinfurthii*	Modern	Gombe	Tanzania	<100BP	2,392,767	2,341,276	10,559	0.451%	108,609	4.639%	104,124	Current Publication
05C	*P*. *t*. *schweinfurthii*	Modern	Gombe	Tanzania	<100BP	4,366,868	4,282,540	17,090	0.399%	151,258	3.532%	104,137	Current Publication
07C	*P*. *t*. *schweinfurthii*	Modern	Gombe	Tanzania	<100BP	11,375,571	11,050,159	60,410	0.547%	633,537	5.733%	104,132	Current Publication
13C	*P*. *t*. *schweinfurthii*	Modern	Gombe	Tanzania	<100BP	22,119,050	21,414,173	112,199	0.524%	1,108,540	5.177%	104,132	Current Publication
14C	*P*. *t*. *schweinfurthii*	Modern	Gombe	Tanzania	<100BP	8,990,777	8,632,541	45,834	0.531%	448,080	5.191%	104,096	Current Publication
16C	*P*. *t*. *schweinfurthii*	Modern	Gombe	Tanzania	<100BP	13,555,409	12,633,888	106,002	0.839%	611,240	4.838%	104,124	Current Publication
17C	*P*. *t*. *schweinfurthii*	Modern	Gombe	Tanzania	<100BP	31,957,595	29,909,915	228,383	0.764%	2,383,910	7.970%	104,107	Current Publication
18C	*P*. *t*. *schweinfurthii*	Modern	Gombe	Tanzania	<100BP	13,803,901	13,434,721	76,516	0.570%	666,058	4.958%	104,114	Current Publication
19C	*P*. *t*. *schweinfurthii*	Modern	Gombe	Tanzania	<100BP	9,842,598	9,625,372	41,364	0.430%	440,133	4.573%	104,090	Current Publication
20C	*P*. *t*. *schweinfurthii*	Modern	Gombe	Tanzania	<100BP	8,038,263	7,174,004	70,588	0.984%	408,387	5.693%	104,110	Current Publication
21C	*P*. *t*. *schweinfurthii*	Modern	Gombe	Tanzania	<100BP	6,327,244	6,163,521	21,301	0.346%	270,917	4.395%	104,113	Current Publication
22C	*P*. *t*. *schweinfurthii*	Modern	Gombe	Tanzania	<100BP	4,816,736	4,250,167	41,539	0.977%	238,235	5.605%	104,081	Current Publication
24C	*P*. *t*. *schweinfurthii*	Modern	Gombe	Tanzania	<100BP	7,196,738	6,385,878	55,875	0.875%	396,794	6.214%	104,143	Current Publication
25C	*P*. *t*. *schweinfurthii*	Modern	Gombe	Tanzania	<100BP	8,853,763	8,690,785	39,893	0.459%	408,207	4.697%	104,119	Current Publication
26C	*P*. *t*. *schweinfurthii*	Modern	Gombe	Tanzania	<100BP	8,915,909	8,509,881	57,197	0.672%	410,290	4.821%	104,133	Current Publication
27C	*P*. *t*. *schweinfurthii*	Modern	Gombe	Tanzania	<100BP	3,700,788	3,241,820	18,158	0.560%	156,372	4.824%	104,105	Current Publication
28C	*P*. *t*. *schweinfurthii*	Modern	Gombe	Tanzania	<100BP	8,348,063	7,808,435	66,066	0.846%	373,363	4.782%	104,105	Current Publication
29C	*P*. *t*. *schweinfurthii*	Modern	Gombe	Tanzania	<100BP	7,012,883	6,693,093	48,113	0.719%	545,857	8.156%	104,105	Current Publication

Oral health in the Gombe chimpanzee population was assessed through examination of both the mandible and maxilla (by R.S.N., with assistance from those mentioned in acknowledgements). A total of 63% (12/19) of chimpanzees exhibited signs of carious and/or abscess lesions with 42% (8/19) possessing afflictions impacting the mandible and 52% (10/19) showing maxillary issues. These numbers represent active caries estimates at the time of death and are likely an underestimate of total lifetime caries, as many teeth were lost throughout the life of the animal. A total of 95% of chimpanzees were observed to have lost at least one tooth across the dental arcade with 74% (14/19) of individuals missing at least one tooth from the mandible and 84% (16/19) of individuals having lost one or more teeth from the maxilla. We compared the presence/absence of caries to genera abundance across chimpanzees and found no significant differences based on presence of active caries/abscesses at time of death. Mann *et al*. did not report AMH oral health states^22^ and although Weyrich *et al*.^9^ reported some dental information from the historic and prehistoric human samples (which were excluded from further analysis) only a single Neanderthal (El Sidrón 1) was reported to have likely suffered from periodontal disease. Thus, there was not enough dental health information to compare these data to data from the Gombe chimpanzee population.

**Figure 1:**
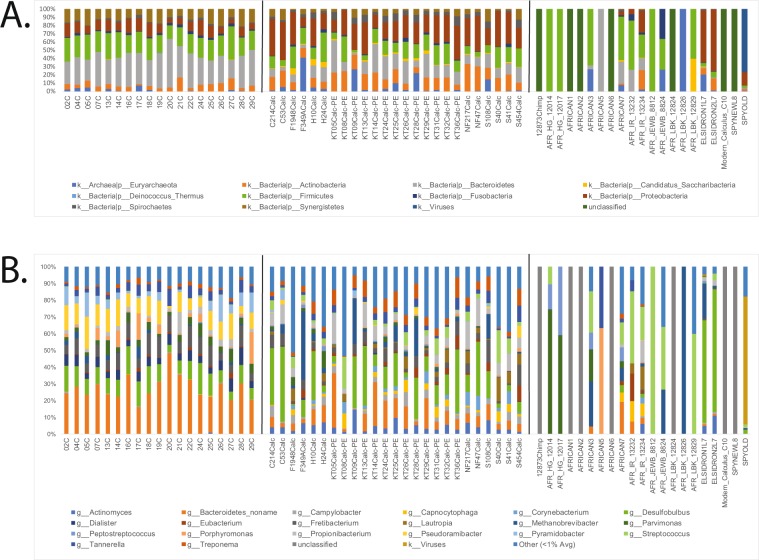
Abundance of sequence reads mapped using MetaPhlAn2 for both (**A**) phyla and (**B**) genera. Leftmost samples are chimpanzees (present study), center samples between black lines are previously published data from Mann *et al*.^22^, and rightmost samples are previously published data from Weyrich *et al*.^9^.

For initial screening purposes, sequences were first compared to the MetaPhlAn2 (metagenomic phylogenetic analysis) database which comprises one million clade-specific marker genes from ~17,000 reference genomes across bacteria, archaea, viruses, and eukaryotes^23^. In both Gombe chimpanzees and historic AMH from Mann *et al*.^22^, samples were dominated by commonly known oral phyla: Actinobacteria, Bacteroidetes, Firmicutes, Proteobacteria, and Synergistetes (Fig. 1). Although the average percentage of reads successfully mapped using MetaPhlAn2 was comparable across populations (0.17% for Weyrich *et al*.^9^, 0.58% for Mann *et al*.^22^, and 0.65% for Gombe chimpanzees), due to the overall low read count of sequences from Weyrich *et al*.^9^, we chose to eliminate all samples aside from the Neanderthals (Spy 1, Spy 2, El Sidrón 1, El Sidrón 2) for downstream analyses.

### Significant phyla and genera using MALT

**Figure 2:**
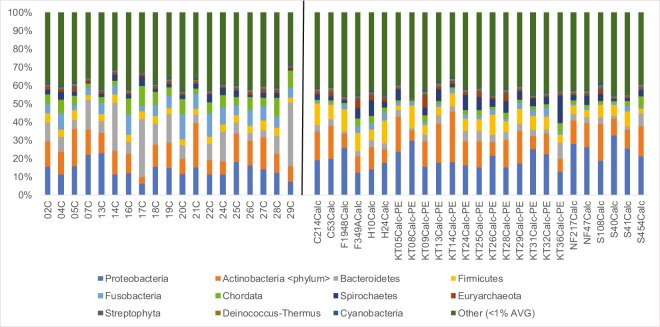
Abundance of sequence reads mapped using MALT for phyla. Leftmost samples are chimpanzees (present study) and rightmost reads are previously published data from Mann *et al*.^22^.

**Figure 3:**
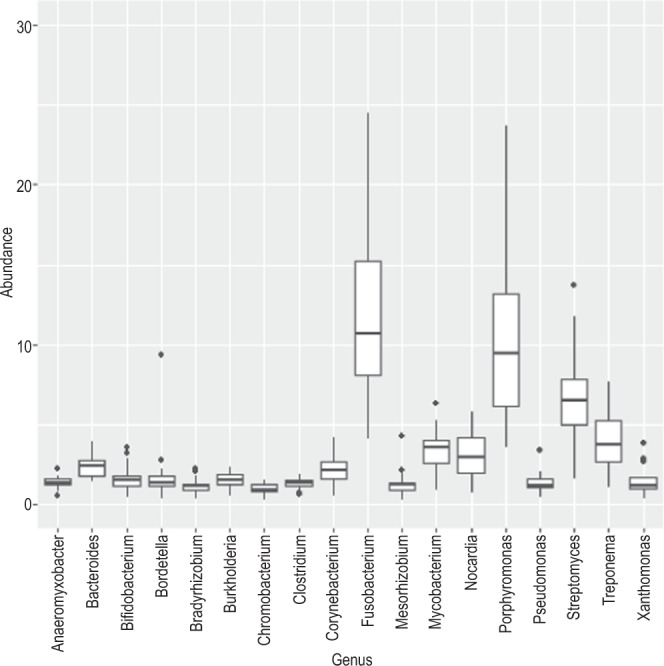
A box plot indicating genera abundance from chimpanzees using MALT. Those individuals (*Porphyromonas* in three chimpanzees) exceeding 30% abundance for any given genus were excluded from the figure for space and clarity purposes.

Mapping with MALT increased the number of reads that mapped to known species since it uses the NCBI nucleotide (or ‘nt’) database (4.82% for Mann *et al*.^22^, and 5.95% for Gombe chimpanzees). Due to the eight (Mann *et al*.^22^) and nine (Gombe) fold increase in mapped reads from MALT compared to MetaPhlAn2 and the extensiveness of the ‘nt’ database compared to MetaPhlAn2, we chose to use the MALT results for subsequent analyses. As such, normalized values (~104,000 reads) from chimpanzees and comparative data were used for downstream analyses. The five most dominant bacterial phyla within the chimpanzee calculus (average across all individuals) are Proteobacteria (22%), Actinobacteria (19.6%), Bacteroidetes (18.7%), Fusobacteria (11.4%), and Firmicutes (6.3%) (Fig. 2). The five most dominant bacterial phyla in AMH (average across all individuals) are Proteobacteria (34.3%), Actinobacteria (21.9%), Firmicutes (12.6%), Spirochaetes (7.6%) and Bacteroidetes (5.8%). A total of four phyla (Table 2) are significantly different between AMH and chimpanzee calculus (above 1% abundance cut off). Bacteroidetes and Fusobacteria are significantly more abundant in chimpanzees, while Firmicutes and Proteobacteria are more dominant in AMH calculus (Kruskal-Wallis, p < 0.05). The five most common bacterial genera in chimpanzees (average across all individuals) are *Porphyromonas* (16.2%), *Fusobacterium* (12%), *Streptomyces* (6.8%), *Treponema* (4%), and *Mycobacterium* (3.4%) (Fig. 3). The five most common bacterial genera in AMH (average across all individuals) are *Treponema* (7.9%), *Streptomyce*s (7.3%), *Neisseria* (7.2%), Streptococcus (6.6%), and *Porphyromonas* (3.6%). Four genera significantly differed between chimpanzees and historic AMH (above 0.5% abundance cut off) (Table 2). *Fusobacterium* and *Porphyromonas* are more abundant within chimpanzees, while *Streptococcus* and *Neisseria* are more common in AMH (all p < 0.05). Hits to both *Pan* and *Homo* (both likely representing host mitogenomes) are present in the sample sets but are not reported here and have been excluded for enterotype analyses.

**Table 2:** Significantly different abundances between chimpanzees and historic anatomically modern humans for both phyla and genera (using MALT, bacteria and archaea only, <0.5% removed).

	Test-Statistic	P	FDR_P	Bonferroni_P	Chimp_mean	Human_mean
**Phyla**						
Bacteroidetes	25.1346	0.0000	0.0000	0.0000	0.1874	0.0582
Fusobacteria	23.9234	0.0000	0.0000	0.0000	0.1139	0.0373
Firmicutes	21.1388	0.0000	0.0000	0.0002	0.0633	0.1259
Proteobacteria	15.8906	0.0001	0.0005	0.0031	0.2196	0.3427
**Genus**						
Streptococcus	25.6274	0.0000	0.0000	0.0002	0.0071	0.0666
Neisseria	24.4043	0.0000	0.0000	0.0004	0.0068	0.0717
Fusobacterium	24.1633	0.0000	0.0000	0.0005	0.1204	0.0384
Porphyromonas	22.0478	0.0000	0.0001	0.0015	0.1617	0.0358

### Enterotype analysis

**Figure 4:**
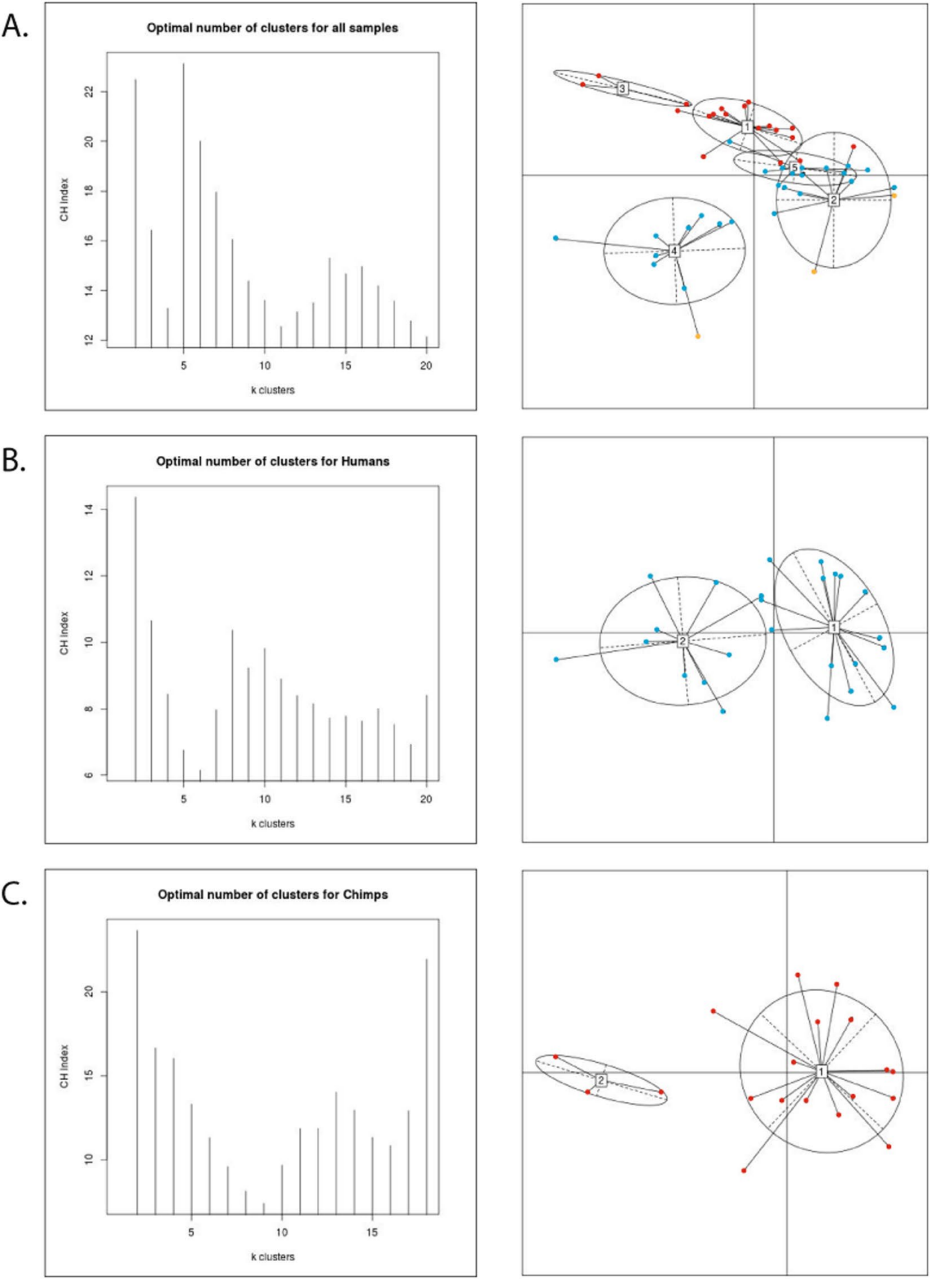
MEGAN normalized (bacteria and archaea only, all zeroes removed) genus level sequence abundance enterotype clustering. The optimal number of clusters and cluster visualization are displayed for (**A**) Neanderthals (Spy1 excluded), anatomically modern humans, and chimpanzees, (**B**) anatomically modern humans only, and (**C**) chimpanzees only. Results are color coded with orange indicating Neanderthals, blue for anatomically modern humans, and red for chimpanzees.

Enterotype analyses (Fig. 4) suggest that chimpanzee and historic AMH samples cluster separately based on the abundance of several core genera. The number of potential clusters for our chosen groupings (AMH/chimpanzees/Neanderthals, chimpanzees only, and AMH only) are estimated using established methods from Arumugam *et al*.^16^. These analyses produced the likely number of sample clusters: five for the AMH/chimpanzees/Neanderthals set, two for the AMH set, and two for the chimpanzee set. Anatomically modern human and chimpanzee clusters are driven by the genera previously mentioned as being significant between the two groups: *Fusobacterium* and *Porphyromonas* (clusters 1 and 2 respectively in Fig. 4C) for chimpanzees, and *Haemophilus* and *Treponema* for AMH (clusters 1 and 2 respectively in Fig. 4B). Neanderthals slightly clustered with historic AMH but the Neanderthal cluster was likely driven by the presence of soil microbiota such as *Arthrobacter* (either modern or ancient) (cluster 2 in Fig. 4A), a potential contaminant noted previously by the authors^9^ (which led to the omission of Spy 1 from enterotype analysis). As such, we cannot conclusively state which genera are driving the clustering of the Neanderthal microbiomes and whether these results are genuine or due to environmental contamination.

### Neighbor joining analyses for microbiomes

**Figure 5:**
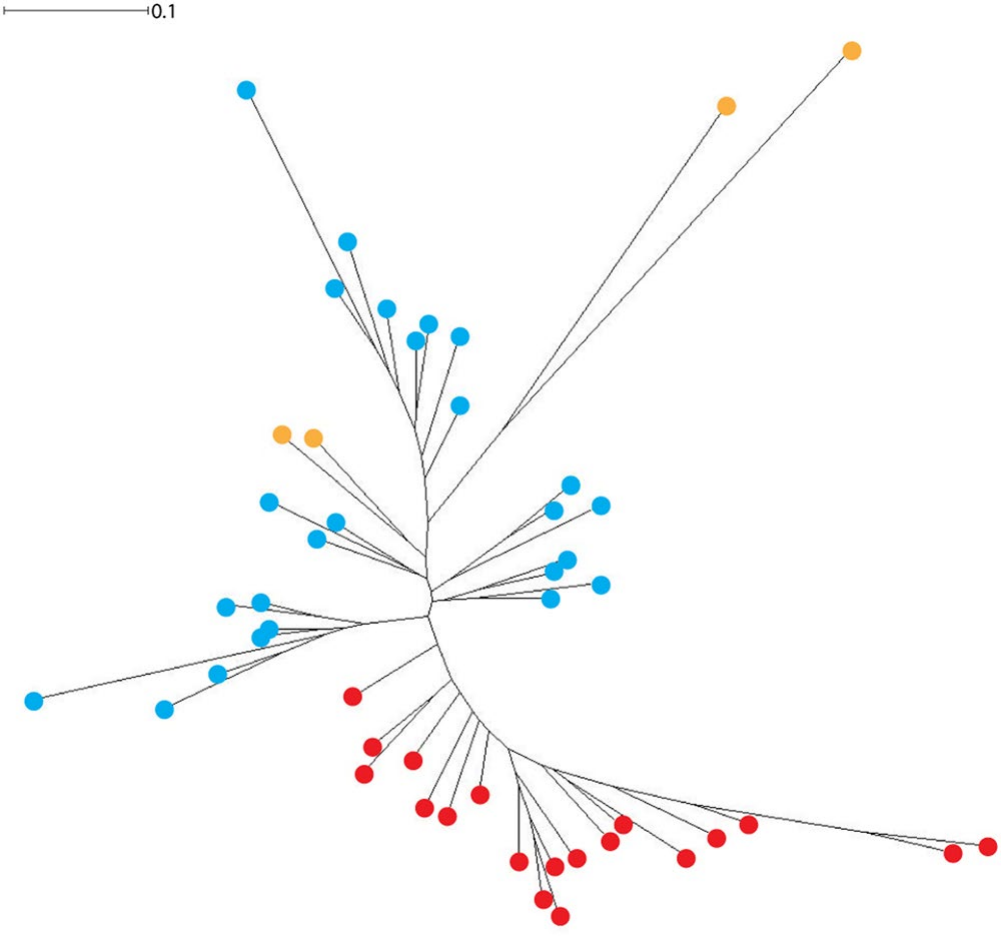
A neighbor joining bray curtis tree using all normalized species in MEGAN (bacteria and archaea only). Results are color coded with orange indicating Neanderthals, blue for anatomically modern humans, and red for chimpanzees.

We used normalized MALT outputs in MEGAN to visualize chimpanzee, Neanderthal, and AMH oral microbiome samples in a Bray Curtis neighbor joining tree (Fig. 5). Neanderthals cluster within the AMH population while chimpanzees cluster separately.

### Red complex analysis

**Figure 6:**
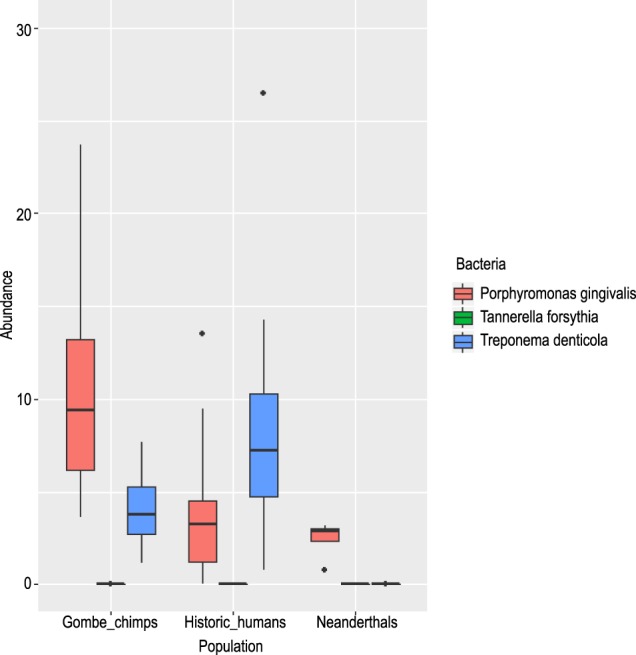
Box plots of normalized species abundance from MEGAN for all three red complex bacteria across Neanderthals, anatomically modern humans, and chimpanzees. Those individuals (*P*. *gingivalis* in three chimpanzees) exceeding 30% abundance for a microbial species were excluded from the figure for space and clarity purposes.

A total of 19 chimpanzee samples, 25 AMH samples^22^, and four Neanderthal samples^9^ were examined for the red complex (using MALTn, normalized in MEGAN) (Fig. 6). Normalized abundance in chimpanzee calculus was an average of 16.2% for *P*. *gingivalis* compared to 3.4% in AMH, which was significant at the p < 0.05 level. Conversely, *T*. *denticola* was more dominant in AMH (7.8%) compared to chimpanzees (4.1%), and this was also significant at the p < 0.005 level. Neanderthal samples showed low read counts of all three members of the red complex, and thus, they were not included in Kruskal-Wallis significance tests. Although we did observe differences in abundances between MetaPhlAn2 and MALT both showed low abundance of *T*. *forsythia* in chimpanzees, which was also shown in a previous study of human dental calculus to be in very high abundance (using MALT)^24^. Additionally, for degraded material, MALT (using BLASTn) was found to be the most accurate method for determining taxonomic information from shotgun sequences^25^.

### Genome reconstruction and phylogenetic tree building

**Figure 7:**
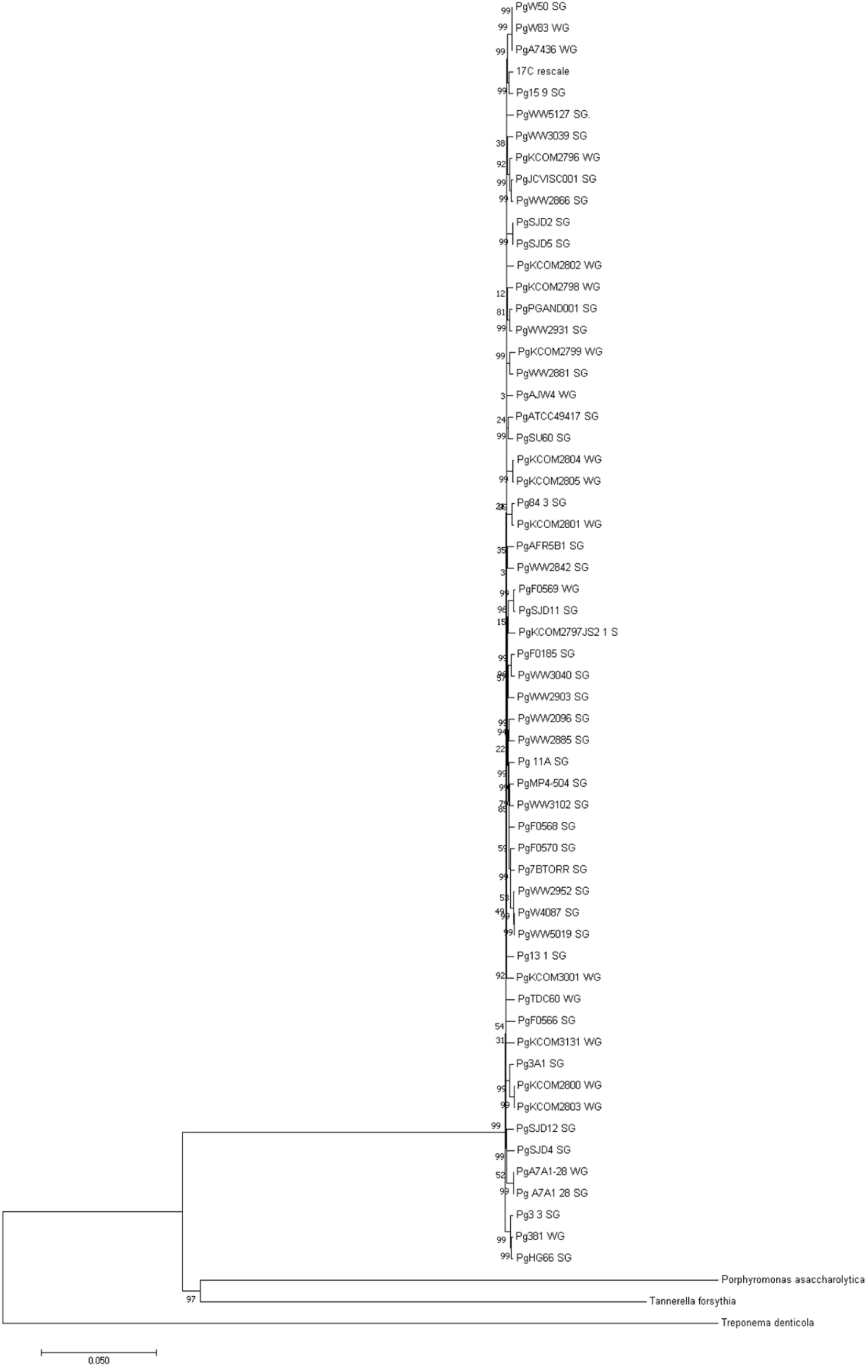
A neighbor joining (500 bootstraps, pairwise deletion) alignment of 58 previously published genomes along with three outgroups: *Porphyromonas asaccharolytica*, *Tannerella forsythia*, *Treponema denticola*.

We used bwa to map dental calculus sequencing reads from the Gombe chimpanzee 17C to the *Porphyromonas gingivalis* genome (NC_010729.1). Out of a total of 29,144,776 merged sequence reads, 838,334 (Q > 30, duplicates removed) reads mapped to *P*. *gingivalis* genome (Supplementary Fig. 1). The GC content of the mapped sequence is slightly less than that of the reference sequence (47.6% compared to 48.4%). A total of 2,118 annotated genes within the *P*. *gingivalis* genome were used for Circos mapping^26^. A total of 2,167,869 bp out of a possible 2,354,886 bp mapped to the reference genome (92.1%). The genome was visualized in 250 bp windows, with a minimum of 0x coverage, a maximum of 123.4x coverage, and an average coverage of 29.2x. This reference aligned genome was compared to 58 previously published *P*. *gingivalis* genomes including three outgroups (*T*. *forsythia*, *T*. *denticola*, *P*. *asaccharolytica*) (Supplementary Table 2). The genome from Gombe did not cluster specifically with those recovered from humans from any one geographic region, with the samples phylogenetically closest originating in Romania, United Kingdom, and United States (Fig. 7).

### Dietary reconstruction

To determine the extent to which DNA sequences recovered from dental calculus showed evidence of host dietary practices (at Q > 20)^27,28^, we used bwa, samtools, and mapDamage 2.0^29,30^. We used 14 full and partial genomes associated with diet analyzed in Weyrich *et al*.^9^ with an additional six genomes from chimpanzee food sources commonly found at Gombe National Park. (Supplementary Tables 3 and 4). In particular, after initial mapping with bwa, we created consensus sequences from five of the seven Neanderthals and from chimpanzee samples. These consensus sequences spanned 11 of the selected dietary genomes (for a total of 22 specific cases of evidence of diet). Our results show that some reads from each individual did map to these dietary reference genomes (0 to 1,355 reads) (Supplementary Table 3). We also examined sequences from our initial MALT analysis that matched each of these species of plants, animals, and fungi (Supplementary Table 4) and found evidence suggesting that some Neanderthal calculus (Spy1 and Spy2) contained traces of *Ovis aries* (sheep) and calculus from one chimpanzee (13C) contained DNA sequences potentially belonging to *Elaeis guineensis* (African palm).

## Discussion

We detected five bacterial phyla in the dental calculus of Gombe chimpanzees (Actinobacteria, Bacteroidetes, Firmicutes, Fusobacteria, and Proteobacteria) which are also commonly found within historic AMH samples. We also found significant differences in abundance between AMH and chimpanzees across four phyla. Previous human calculus studies^8,10^ showed a high abundance of Firmicutes and Proteobacteria, and we report that these phyla are significantly reduced in the mouths of chimpanzees. Conversely, chimpanzees had significantly higher Bacteroidetes and Fusobacteria compared to historic AMH. Actinobacteria, another phylum reported as being abundant in the human oral cavity^8,10^ was also abundant in chimpanzees, but not to a significant degree over historic AMH. Additionally, we report a number of abundant genera in chimpanzee dental calculus including *Fusobacterium*, *Porphyromonas*, and *Streptomyces* (>5% average abundance). Both *Fusobacterium* and *Porphyromonas* abundance was significantly higher in chimpanzees compared to historic AMH (p < 0.05). The significance of *Fusobacterium* within the oral cavity is not fully understood. In some studies it was reported that *Fusobacterium* could be either a pathogen or commensal^31^, while others show associations with oral disease and systemic issues throughout the body^32^. It should be noted that the mere presence of a genus that contains pathogenic species does not mean the species found here play a pathogenic role in the oral cavity. Our analysis of chimpanzee oral health based on caries and tooth loss did not find a significant association between one particular genus and the presence of caries or the absence of teeth. In addition to questions about the role of these bacteria in health states, it should also be noted that these differences seen between AMH and chimpanzees may stem from environmental differences. Comparative AMH samples from Mann *et al*.^22^ were from several locations across Asia, Europe, and North America, while our chimpanzee data only represent Gombe National Park, one location in Eastern Africa. Future studies sampling historic nonhuman primates and human populations in Africa may show similar oral microbiome signatures to those recovered from wild chimpanzees from Gombe National Park.

Our data show that oral microbiomes from AMH, chimpanzees, and Neanderthals did not adhere to an enterotype clustering pattern reminiscent of the gut microbiome. A global study of human gut metagenomes found that individuals cluster into three robust enterotype groups that are independent of body mass index, age, gender, and geographic location^16^. Our results however, do not necessarily cluster randomly as seen in previous studies^16^, but somewhat along host species lines, with most chimpanzees clustering together, most AMH clustering together, and a with smaller group of AMH and Neanderthals set slightly apart. The driving genera are those noted as being significantly different between AMH and chimpanzees. Specifically, AMH enterotypes are driven by *Treponema*, which has strong associations with periodontal disease^33^ and *Haemophilus* which can be commonly found in human plaque^34^ and has been associated with a healthy human mouth^35^. However, species of *Haemophilus* also exhibit pathogenic properties throughout the body^36^. Secondary drivers of these AMH enterotypes include both *Streptococcus*, which has been identified as both a genus including commensal and pathogenic species^37^, and *Neisseria*, which also exhibits both pathogenic and non-pathogenic strains in humans^38^. The signature in the Neanderthal calculus seems to be driven by *Arthrobacter*, which is a common soil microbe^39^ but has also associated with skin lesions in humans^40^. Chimpanzee enterotypes were driven by both *Fusobacterium* and *Porphyromonas*, both of which are considered by some to be causative agents in periodontal disease^41^. Unfortunately, we do not have oral health data from the archaeological samples sequenced by Mann *et al*.^22^, and there was not a significant difference in abundance of *Fusobacterium* and *Porphyromonas* related to caries or tooth loss in chimpanzees. Independent of health states, the partitioning of these enterotypes by host species echoes what was observed in previous studies of human and chimpanzee salivary microbiomes^15^. In the years since enterotypes were first proposed, they were found to be associated with long-term diet^42^ and population^43^, with some studies suggesting enterotypes are not as distinct as first documented^44^ and others questioning the existence of discrete clusters completely^45^. For example, a subsequent study examined how sample processing and data analysis can alter enterotype recovery, but note that enterotypes are still beneficial for exploring overall microbial composition^19^. Here we use the original definition of enterotypes to investigate primate dental calculus microbiomes and show that they mainly adhere to a two-group system (based on host species). We posit that both AMH and chimpanzee clusters are likely driven by long term unhealthy oral states within the host as reflected in the increased abundance of known pathogens belonging to the genera *Porphyromonas* and *Fusobacterium* in chimpanzees and *Haemophilus* and *Treponema* in AMH.

A known cause of oral dysbiosis within humans is attributed to periodontal disease^46^. This disease is commonly associated with pathogenic microbiota collectively referred to as the red complex (*Porphyromonas gingivalis*, *Treponema denticola*, *Tannerella forsythia* (formerly *Bacteroides forsythus*)). Initially the detection of red complex bacteria was linked to poor oral health^5^ but it is by no means the only indicator of periodontal disease^47^. Observable traits in skeletal remains including, tooth loss, tooth wear, and abscesses are manifestations of periodontal infection and have been documented in captive and wild great apes^48,49^, but the connection between these and the red complex bacteria in the *Pan* oral cavity is not known. Studies have shown both positive and negative correlations between the presence of *P*. *gingivalis* and oral disease states^50–52^ yet others suggest their abundance is independent of disease and more closely related to host weight^53^ and age^54–56^. However, species of *Porphyromonas* likely have different roles within the mouth at different times^57^ with *P*. *gingivalis* acting as a late colonizer which inhabits the top layer of already formed biofilms^58^ and a species such as *P*. *catoniae* occupying the mouths of infants prior to tooth eruption^59^. In longitudinal studies, the abundance of *T*. *denticola* and *P*. *gingivalis* are linked together as indicators of chronic periodontitis progression^60^. However, our results suggest that their increased abundance is not always linked, due to the low presence of *T*. *denticola* across chimpanzees. Low abundance of *Tannerella* was also reported in the oral cavity of another nonhuman primate, Rhesus macaques (*Macaca mulatta*)^61^ from the Caribbean Primate Research Center in Puerto Rico. Although we observed caries and abscesses within the dental arcade of several chimpanzees, we cannot make statements regarding the role of any single microbe or any group of microbes as causative agents of disease. It is likely a very complex process involving many elements, as dental calculus recovered from healthy human teeth and those afflicted with periodontal disease do not significantly differ in microbial, protein, and metabolomic profiles^62^. As such, it is imperative to continue to characterize oral microbiomes from modern and historic primates with varying health states in order to further comprehend the factors that drive these complex ecosystems.

The *Porphyromonas gingivalis* genome recovered from one of the Gombe chimpanzees was selected for analysis because it was the most complete genome observed with the highest level of total coverage. The phylogenetic analyses of a *P*. *gingivalis* genome assembled from a single chimpanzee individual (17C) did not distinctly separate it from previously published genomes. However, research suggests that *P*. *gingivalis* strains likely undergo frequent recombination with other strains^63^ which may obscure phylogeography. These DNA exchange events generates diverse phenotypes among microbial communities^64^. In *P*. gingivalis, the high mosaicity arises from an increase in the likelihood of recombination events due to the use of carbon from exogenous DNA as sources of energy^63,65^. Considering that *P*. *gingivalis* has a complex genome that readily recombines, it would be beneficial in the future to isolate, culture, and sequence this microbe in chimpanzee plaque using traditional laboratory methods in order to understand the nuanced differences in genotypes and phenotypes of this strain.

Because the chimpanzees at Gombe have been subject to decades of observation^20,21,66,67^, their diet is known and this can be used to assess whether dental calculus preserves genetic material from plants and animals indicative of dietary habits. We searched for evidence of dietary DNA sequences in five Neanderthal samples and two Gombe chimpanzees using full and partial genome reference data from fourteen organisms (Weyrich *et al*.^9^) and an additional six associated with the environment in Gombe National Park. Although some short sequences mapped to possible dietary sources (Supplementary Table 3), an additional screening of the initial MALT results show only two cases in which dietary DNA may be present: sheep sequences in the Spy Neanderthals and palm DNA in one of the Gombe chimpanzees. Although it is not out of the realm of possibility that dietary DNA is present in these and Weyrich *et al*.^9^ calculus samples, due to the very nature of ancient and degraded historic DNA (short fragments), the lack of high sequencing depth, and the presence of only highly conserved regions in 16S ribosomal RNA genes and chloroplast DNA in most reference databases, we hesitate to conclude that these sequences definitively originate from the hosts’ diet. We suggest that future dietary analyses use proteomics and phytoliths along with genome capture in order to confirm shotgun DNA sequence data. Additionally, we stress using caution when interpreting ‘shared’ oral microbial genera as being indicative of ‘interaction’ between individuals, in agreement with other authors^68^.

In conclusion, our results present an important piece of the puzzle in understanding the composition and evolution of the primate oral microbiome. Chimpanzee and AMH oral microbes differ significantly but it is still unclear as to the underlying causes of these differences: diet, geography, host genomes, or factors unknown. Future studies should continue to integrate bioarchaeological, observational, and cultural evidence into studies of historic microbiomes whenever possible in order to establish the most complete picture of primate oral ecologies.

## Materials and Methods

### Sample collection and extraction

A total of 19 calculus samples were removed from Gombe chimpanzee skeletal remains. The source of the chimpanzee skeletal remains is the long-term non-invasive study led by Dr. Jane Goodall. No chimpanzees were harmed to obtain these skeletal remains. Bodies of chimpanzees that died from natural causes were recovered and either buried or kept in a container until soft tissues had decayed^69^. Due to the lack of abundant calculus across the dental arcade of Gombe chimpanzees, samples were collected opportunistically and pooled together for each single individual. When available, calculus was sampled from at least one tooth on both the mandibular and maxillary sides (<15 mg total). Overall dental health was also assessed at the time of sampling (Supplementary Table 1). Teeth were counted as having a carious lesion if the enamel was infiltrated and not caused by a clear breakage (many of the teeth are discolored, making a true assessment of cavities difficult). Teeth with abscesses also qualified as carious lesions. Tooth loss was classified as a clear resorption of bone and not caused by postmortem damage (marked with ‘O’ for adult teeth and ‘dO’ for deciduous teeth).

Samples were shipped to a UV-equipped, class 10,000 HEPA filtration ancient DNA facility at Arizona State University. Throughout the preparation and extraction of specimens, full ancient lab precautions were utilized including full length sterile suits, hairnets, facemasks, and eye protection. Calculus samples were pulverized using a sterile hammer and UV-ed in a DNA crosslinker for 2 minutes on each side (5– 15 mg). Samples were transferred to a 2 mL tube and washed using 1 mL of 0.5 M EDTA (Ambion) on a rotating nutator for 15 minutes at room temperature (RT). They were then centrifuged at 13.2 k rpm for 3 minutes and the supernatant was removed and discarded. Fresh EDTA (1 mL) was added to the pellet and resuspended by vortexing and placed on a rotating nutator overnight at RT. A total of 100 µL of Proteinase K (Qiagen) was added to the 2 mL tube and set on a rotating nutator at 37 °C for 8 hours. Samples were left to rotate overnight at RT once more. The next day samples were centrifuged at 13.2 k rpm for 3 minutes and the supernatant was kept at 4 °C. Fresh EDTA was added to the pellet along with 50 µL more of Proteinase K. Samples were left to rotate overnight one final time at RT. Samples were centrifuged at 13.2 k rpm for 3 minutes and both supernatants were added to a total of 12 mL of PB Buffer (Qiagen) in a Zymo reservoir attached to a MinElute PCR Purification kit (Qiagen) silica column (within a 50 mL Falcon tube). Samples were spun for 6 k rpm for 4 minutes, rotated 180° and spun another 2 minutes. The MinElute column was washed according to manufacturer specifications and eluted into 30 µL.

### Shotgun build, amplification, and sequencing

Extracts for calculus samples underwent double stranded shotgun builds. For initial blunt end repair, a total of 20 µL (~800 ng) of DNA was added to 5.0 µL NEB Buffer, 0.50 µL dNTP mix (2.5 mM), 4.0 µL BSA (10 mg/mL), 5.0 µL ATP (10 mM), 2.0 µL T4 PNK, 0.40 µL T4 Polymerase, and 13.10 µL ddH_2_O was incubated at 15 °C for 15 minutes followed by 25 °C for 15 minutes. The solution was then purified using a MinElute according to manufacturer protocol and eluted into 18 µL EB buffer. For adapter ligation, 18 µL of template DNA was added to 20 µL Quick Ligase Buffer, 1.0 µL Solexa Mix^70^, and 1.0 µL Quick Ligase and incubated at room temperature for 20 minutes. The solution was then purified again using a MinElute according to manufacturer protocol and eluted into 20 µL EB buffer. For the final fill in portion of the shotgun build, 20 µL of template DNA was added to 4.0 µL Thermo pol buffer, 0.50 µL dNTP mix (2.5 mM), 2.0 µL Bst polymerase, and 13.50 µL ddH_2_O was incubated at 37 °C for 20 minutes followed by 80 °C for 20 minutes. Following shotgun preparation, samples were amplified using Amplitaq Gold DNA Polymerase (Thermo Fisher Scientific) to a total of 10 cycles. Shotgun libraries were split into four identical PCR reactions which contained 9.0 µL of DNA, 9.27 µL PCR Buffer II (10x), 9.27 µL MgCl_2_ (25 mM), 3.68 µL dNTP mix (10 nM), 2.21 µL BSA (10 mg/mL), 2.0 µL P5 primer, 2.0 µL P7 primer, 61.09 µL of ddH_2_O, and 1.48 µL of Amplitaq Gold enzyme. The PCR conditions were as follows: initial denaturation at 95 °C for 15 minutes, followed by cycling of 95 °C for 30 seconds, 58 °C for 30 seconds, and 72 °C for 45 seconds, with a final elongation of 72 °C for 10 minutes. Each P5 and P7 primer pair used for the four samples had a unique set of barcodes^71^ in order to separate the individual samples from the pooled material bioinformatically. Samples were purified using the MinElute according to manufacturer protocol and eluted into 30 µL of EB buffer. After checking concentration using a DNA1000 Bioanalyzer chip (Agilent) samples were pooled in equimolar amounts and pooled on a single Illumina HiSeq. 2500 2 × 100 pe (Rapid Mode) lane at the Yale Center for Genome Analysis (YCGA). Two of the chimpanzee samples were sequenced deeper (13C and 17C) with chimpanzee exome captures a sequencing run with the same specifications at YCGA.

### Sequence processing and data analysis

Samples for this publication were returned as de-multiplexed reads from YGCA and paired end samples from comparative studies were downloaded from the Online Ancient Gene Repository (OAGR) under the project title “Reconstructing Neanderthal behavior, diet, and disease using ancient DNA from dental calculus” (https://www.oagr.org.au/experiment/view/65/) for Weyrich *et al*.^9^ and the NCBI Short Read Archive (SRA) under the Bioproject accession PRJNA445215 (https://www.ncbi.nlm.nih.gov/bioproject/PRJNA445215/) for Mann *et al*.^22^. For the chimpanzee sample set in the present study, Weyrich *et al*.^9^, and Mann *et al*.^22^, paired end files were unzipped, adapters were removed, and paired ends were merged using SeqPrep^72^ with a minimum overlap of 30 bp and a minimum quality threshold of 20. Taxonomic abundances of phyla and genera were inferred using MetaPhlAn2.0^73^, as used in previous publications^74^. Additionally, reads were mapped to the NCBI nucleotide database using MALT (BLASTn (February 2017), 85% sequence similarity, minimum support percent of 0.01, top percent value of 1.0)^75^ and analyzed in MEGAN^76^. MALT analyses were carried out using XSEDE^77^. MEGAN allowed the data to be normalized and grouped into shared species using a bray Curtis neighbor joining method (only bacteria and archaea selected). We used normalized abundance (Table 1) from MEGAN to determine the totals of phyla and genera across samples. We used Kruskal-Wallis within R to determine significant phyla and genera between human and chimpanzee groups^78^. For enterotyping, we used normalized count data from all three groups (Neanderthals, AMH, and chimpanzees) and used methods from a previous publication^16^ to call clusters and generate figures within R. Spy 1 was removed from Fig. 4A due to contamination concerns presented by Weyrich *et al*.^9^

Prior to mapping, raw reads from 17C were adapter trimmed and merged using seqprep (>Q30)^72^. Reads were mapped to the *Porphyromonas gingivalis ATCC 33277* genome (NC_010729.1)^79^ using BWA v. 0.7.5^27^ following recommendations by Schubert *et al*.^80^. Mapped reads were quality filtered (>Q30), duplicates were removed, and sequences with multiple mappings were removed using Samtools v. 0.1.19^28^. The program mapDamage 2.0 was used to rescale BAM files and characterize damage patterns^29,30^. The full genome was visualized in Geneious 9^81^ (https://www.geneious.com/) which was used to export a consensus sequence. The consensus sequence was visualized using Circos^26^ with gray bars indicating 25x to 125x coverage (intervals of 25) and each green line extending outward representing a 250 bp window of base pair coverage. Total coverage is represented by the inner green coloration (250 bp windows), and GC content represented by a second green circle (250 bp windows) with a gray line representing average GC content.

A total of 58 full and partially assembled genomes from *P*. *gingivalis* (ftp://ftp.ncbi.nlm.nih.gov/genomes/genbank/bacteria/Porphyromonas_gingivalis/latest_assembly_versions/) were downloaded from Genbank and the sequences were aligned to the reference genome using previously published methods^82^ (Supplementary Table 1). In brief, for each previously published complete or partial genomes, we used similar methods to those reported for 17C (using BWA v. 0.7.5^27^ and Samtools v. 0.1.19^28^ but not mapDamage 2.0^29,30^). Then using Picard^83^, a sequence dictionary was created with the aforementioned reference genome for *Porphyromonas gingivalis*. Lastal^84^ and Samtools v. 0.1.19^28^ were used to convert each mapped genome to sam and bam files, and bcftools^85^ was used to create a VCF file. GATK^86^ was then used to combine variants from all files and custom scripts were used to create a VCF variant table and finally a FASTA alignment. The resulting file was used to create a neighbor joining tree (500 bootstraps) using MEGA7^87^.

Previously published full and partial genomes indicative of diet (Supplementary Tables 3 and 4) were downloaded from NCBI. We mapped two chimpanzee samples 13C and 17C (due to their high sequencing depth) and four samples from Spy and El Sidrón (including an additional deeper sequenced El Sidrón 1 sample labelled merely ‘ELSIDRON’) against 15 indicators of diet present in Weyrich *et al*.^9^ along with six additional indicators of diet that documented in observational data compiled from Gombe National Park^20,21^. We selected several commonly eaten items, but it should be noted that some foods are eaten during restricted fruiting seasons and not necessarily year round^20,21^. We used identical methods to those used to map the 17C *P*. *gingivalis* genome but reduced the quality filtering during seqprep and mapping to 20. The number of reads that mapped to their dietary species are reported in Supplementary Table 3. Additionally, we compiled raw reads from the original MALT analysis that matched these dietary sources and reported those values in Supplementary Table 4.

## Supplementary information

Supplemental Figure 1

Supplemental Tables 1–4

## Data Availability

Raw data sequences have been deposited into the NCBI Short Read Archive (SRA) under the BioProject ID PRJNA531027 (SAMN11408660-SAMN11408678).

## References

[1] Dewhirst FE (2010). The Human Oral Microbiome. Journal of Bacteriology.

[2] Nasidze I, Li J, Quinque D, Tang K, Stoneking M (2009). Global diversity in the human salivary microbiome. Genome Research.

[3] Mason MR, Nagaraja HN, Camerlengo T, Joshi V, Kumar PS (2013). Deep Sequencing Identifies Ethnicity-Specific Bacterial Signatures in the Oral Microbiome. Plos One.

[4] Yang F (2012). Saliva microbiomes distinguish caries-active from healthy human populations. The ISME Journal.

[5] Socransky SS, Haffajee AD, Cugini MA, Smith C, Kent RL (1998). Microbial complexes in subgingival plaque. Journal of Clinical Periodontology.

[6] Socransky SS, Haffajee AD (2005). Periodontal microbial ecology. Periodontology 2000.

[7] Ozga AT (2016). Successful enrichment and recovery of whole mitochondrial genomes from ancient human dental calculus. American Journal of Physical Anthropology.

[8] Adler CJ (2013). Sequencing ancient calcified dental plaque shows changes in oral microbiota with dietary shifts of the Neolithic and Industrial revolutions. Nat Genet.

[9] Weyrich LS (2017). Neanderthal behaviour, diet, and disease inferred from ancient DNA in dental calculus. Nature.

[10] Warinner C (2014). Pathogens and host immunity in the ancient human oral cavity. Nature genetics.

[11] Santiago-Rodriguez TM, Narganes-Storde Y, Chanlatte-Baik L, Toranzos GA, Cano RJ (2017). Insights of the dental calculi microbiome of pre-Columbian inhabitants from Puerto Rico. PeerJ.

[12] Nasidze I (2011). High diversity of the saliva microbiome in Batwa Pygmies. PLoS One.

[13] Contreras M (2010). The bacterial microbiota in the oral mucosa of rural Amerindians. Microbiology.

[14] Ozga Andrew T., Sankaranarayanan Krithivasan, Tito Raúl Y., Obregon-Tito Alexandra J., Foster Morris W., Tallbull Gloria, Spicer Paul, Warinner Christina G., Lewis Cecil M. (2016). Oral microbiome diversity among Cheyenne and Arapaho individuals from Oklahoma. American Journal of Physical Anthropology.

[15] Li J (2013). The saliva microbiome of Pan and Homo. BMC Microbiology.

[16] Arumugam M (2011). Enterotypes of the human gut microbiome. Nature.

[17] Moeller AH (2012). Chimpanzees and Humans Harbor Compositionally Similar Gut Enterotypes. Nature communications.

[18] Moeller AH (2015). Stability of the Gorilla Microbiome Despite SIV Infection. Molecular ecology.

[19] Costea PI (2018). Enterotypes in the landscape of gut microbial community composition. Nature Microbiology.

[20] van Lawick-Goodall J (1968). The behaviour of free-living chimpanzees in the Gombe Stream Reserve. Animal Behaviour Monographs.

[21] Wrangham, R. W. In *Primate Ecology: Studies of feeding and ranging behaviour in lemurs*, *monkeys and apes* (ed. Clutton-Brock, T. H.) Ch. 17, 503–538 (Academic Press, 1977).

[22] Mann AE (2018). Differential preservation of endogenous human and microbial DNA in dental calculus and dentin. Scientific Reports.

[23] Truong DT (2015). MetaPhlAn2 for enhanced metagenomic taxonomic profiling. Nature Methods.

[24] Willmann C (2018). Oral health status in historic population: Macroscopic and metagenomic evidence. PLoS ONE.

[25] Eisenhofer R, Weyrich LS (2019). Assessing alignment-based taxonomic classification of ancient microbial DNA. PeerJ.

[26] Krzywinski M (2009). Circos: an information aesthetic for comparative genomics. Genome Res.

[27] Li H, Durbin R (2009). Fast and accurate short read alignment with Burrows–Wheeler transform. Bioinformatics.

[28] Li H (2009). The Sequence Alignment/Map format and SAMtools. Bioinformatics.

[29] Ginolhac Aurelien, Rasmussen Morten, Gilbert M. Thomas P., Willerslev Eske, Orlando Ludovic (2011). mapDamage: testing for damage patterns in ancient DNA sequences. Bioinformatics.

[30] Jonsson H, Ginolhac A, Schubert M, Johnson PL, Orlando L (2013). mapDamage2.0: fast approximate Bayesian estimates of ancient DNA damage parameters. Bioinformatics.

[31] Signat B, Rogues C, Poulet P, Duffaut D (2011). Fusobacterium nucleatum in periodontal health and disease. Current issues in molecular biology.

[32] Han YW (2015). Fusobacterium nucleatum: a commensal-turned pathogen. Current opinion in microbiology.

[33] Dashper SG, Seers CA, Tan KH, Reynolds EC (2011). Virulence factors of the oral spirochete Treponema denticola. Journal of dental research.

[34] Liljemark WF (1984). Distribution of oral Haemophilus species in dental plaque from a large adult population. Infection and immunity.

[35] Bik EM (2010). Bacterial diversity in the oral cavity of 10 healthy individuals. ISME J.

[36] King P (2012). Haemophilus influenzae and the lung (Haemophilus and the lung). Clinical and translational medicine.

[37] Abranches, J. *et al*. Biology of Oral Streptococci. *Microbiology**Spectrum* **6**, 10.1128/microbiolspec.GPP3-0042-2018 (2018). 10.1128/microbiolspec.gpp3-0042-2018 PMC6287261 30338752

[38] Liu G, Tang CM, Exley RM (2015). Non-pathogenic *Neisseria*: members of an abundant, multi-habitat, diverse genus. Microbiology.

[39] Mongodin EF (2006). Secrets of Soil Survival Revealed by the Genome Sequence of Arthrobacter aurescens TC1. PLOS Genetics.

[40] Huang Y (2005). Arthrobacter scleromae sp. nov. isolated from human clinical specimens. Journal of clinical microbiology.

[41] Han YW (2000). Interactions between Periodontal Bacteria and Human Oral Epithelial Cells: *Fusobacterium nucleatum* Adheres to and Invades Epithelial Cells. Infection and immunity.

[42] Wu GD (2011). Linking long-term dietary patterns with gut microbial enterotypes. Science.

[43] Ou J (2013). Diet, microbiota, and microbial metabolites in colon cancer risk in rural Africans and African Americans. The American Journal of Clinical Nutrition.

[44] Huse SM, Ye Y, Zhou Y, Fodor AA (2012). A Core Human Microbiome as Viewed through 16S rRNA Sequence Clusters. PLoS ONE.

[45] Knights D (2014). Rethinking “Enterotypes”. Cell host & microbe.

[46] Sundqvist G, Figdor D (2003). Life as an endodontic pathogen. Endodontic Topics.

[47] Arora N, Mishra A, Chugh S (2014). Microbial role in periodontitis: Have we reached the top? Some unsung bacteria other than red complex. Journal of Indian Society of Periodontology.

[48] Legge SS (2012). Dentoalveolar abscess variation among three groups of chimpanzees (Pan troglodytes schweinfurthii, Pan troglodytes troglodytes, and Pan paniscus). International Journal of Paleopathology.

[49] Lowenstine LJ, McManamon R, Terio KA (2016). Comparative Pathology of Aging Great Apes: Bonobos, Chimpanzees, Gorillas, and Orangutans. Veterinary Pathology.

[50] Frandsen EVG, Poulsen K, Curtis MA, Kilian M (2001). Evidence of Recombination in *Porphyromonas gingivalis* and Random Distribution of Putative Virulence Markers. Infection and immunity.

[51] Rôças IN, Siqueira JF, Santos KRN, Coelho AMA, de Janeiro R (2001). “Red complex” (Bacteroides forsythus, Porphyromonas gingivalis, and Treponema denticola) in endodontic infections: A molecular approach. Oral Surgery, Oral Medicine, Oral Pathology, Oral Radiology and Endodontics.

[52] Ximenez-Fyvie LA, Haffajee AD, Socransky SS (2000). Comparison of the microbiota of supra- and subgingival plaque in health and periodontitis. J Clin Periodontol.

[53] Matsushita K (2015). The novel association between red complex of oral microbe and body mass index in healthy Japanese: a population based cross-sectional study. Journal of Clinical Biochemistry and Nutrition.

[54] Savitt ED, Kent RL (1991). Distribution of Actinobacillus actinomycetemcomitans and Porphyromonas gingivalis by Subject Age. Journal of periodontology.

[55] Umeda M (1998). Risk Indicators for Harboring Periodontal Pathogens. Journal of periodontology.

[56] Papaioannou W (2009). The microbiota on different oral surfaces in healthy children. Oral microbiology and immunology.

[57] Darveau RP (2009). The Oral Microbial Consortium’s Interaction with the Periodontal Innate Defense System. DNA and Cell Biology.

[58] Zijnge V (2010). Oral Biofilm Architecture on Natural Teeth. PLoS One.

[59] Könönen E, Kanervo A, Takala A, Asikainen S, Jousimies-Somer H (1999). Establishment of Oral Anaerobes during the First Year of Life. Journal of dental research.

[60] Byrne SJ (2009). Progression of chronic periodontitis can be predicted by the levels of Porphyromonas gingivalis and Treponema denticola in subgingival plaque. Oral microbiology and immunology.

[61] Kirakodu, S., Chen, J., Gonzalez Martinez, J., Gonzalez, O. A. & Ebersole, J. Microbiome Profiles of Ligature-Induced Periodontitis in Nonhuman Primates Across the Lifespan. *Infection and immunity*, IAI.00067–00019, 10.1128/iai.00067-19 (2019). 10.1128/IAI.00067-19 PMC6529664 30885927

[62] Velsko IM (2019). Microbial differences between dental plaque and historic dental calculus are related to oral biofilm maturation stage. Microbiome.

[63] Tribble, G. D. *et al*. Natural Competence Is a Major Mechanism for Horizontal DNA Transfer in the Oral Pathogen Porphyromonas gingivalis. *mBio* **3**, 10.1128/mBio.00231-11 (2012). 10.1128/mBio.00231-11 PMC3268665 22294679

[64] Tribble GD, Kerr JE, Wang B-Y (2013). Genetic diversity in the oral pathogen Porphyromonas gingivalis: molecular mechanisms and biological consequences. Future microbiology.

[65] Acuña-Amador L, Primot A, Cadieu E, Roulet A, Barloy-Hubler F (2018). Genomic repeats, misassembly and reannotation: a case study with long-read resequencing of Porphyromonas gingivalis reference strains. BMC Genomics.

[66] White FJ, Wrangham RW (1988). Feeding competition and patch size in the chimpanzee species *Pan paniscus* and *Pan troglodytes*. Behaviour.

[67] Goodall, J. *The Chimpanzees of Gombe: Patterns of Behavior*. (Harvard University Press, 1986).

[68] Charlier P, Gaultier F, Héry-Arnaud G (2018). Interbreeding between Neanderthals and modern humans: Remarks and methodological dangers of a dental calculus microbiome analysis. Journal of Human Evolution.

[69] Kirchhoff CA (2018). Infanticide in chimpanzees: Taphonomic case studies from Gombe. American Journal of Physical Anthropology.

[70] Meyer M., Kircher M. (2010). Illumina Sequencing Library Preparation for Highly Multiplexed Target Capture and Sequencing. Cold Spring Harbor Protocols.

[71] Kircher M, Sawyer S, Meyer M (2012). Double indexing overcomes inaccuracies in multiplex sequencing on the Illumina platform. Nucleic acids research.

[72] SeqPrep, https://github.com/jstjohn/SeqPrep.

[73] Segata N (2012). Metagenomic microbial community profiling using unique clade-specific marker genes. Nature Methods.

[74] Tung J (2015). Social networks predict gut microbiome composition in wild baboons. eLife.

[75] Herbig, A. *et al*. MALT: Fast alignment and analysis of metagenomic DNA sequence data applied to the Tyrolean Iceman. *bioRxiv*, 10.1101/050559 (2016).

[76] Huson DH, Auch AF, Qi J, Schuster SC (2007). MEGAN analysis of metagenomic data. Genome Research.

[77] Towns J (2014). XSEDE: Accelerating Scientific Discovery. Computing in Science & Engineering.

[78] R: A Language and Environment for Statistical Computing (R Foundationg for Statistical Computing, Vienna, Austria, 2014).

[79] Naito M (2008). Determination of the Genome Sequence of Porphyromonas gingivalis Strain ATCC 33277 and Genomic Comparison with Strain W83 Revealed Extensive Genome Rearrangements in P. gingivalis. DNA Research: An International Journal for Rapid Publication of Reports on Genes and Genomes.

[80] Schubert M (2014). Characterization of ancient and modern genomes by SNP detection and phylogenomic and metagenomic analysis using PALEOMIX. Nature Protocols.

[81] Geneious_9, https://www.geneious.com, https://www.geneious.com

[82] Honap TP (2018). Mycobacterium leprae genomes from naturally infected nonhuman primates. PLoS neglected tropical diseases.

[83] PicardTools (2018).

[84] Kiełbasa SM, Wan R, Sato K, Horton P, Frith MC (2011). Adaptive seeds tame genomic sequence comparison. Genome Research.

[85] Li H (2011). A statistical framework for SNP calling, mutation discovery, association mapping and population genetical parameter estimation from sequencing data. Bioinformatics (Oxford, England).

[86] McKenna A (2010). The Genome Analysis Toolkit: a MapReduce framework for analyzing next-generation DNA sequencing data. Genome research.

[87] Kumar S, Stecher G, Tamura K (2016). MEGA7: Molecular Evolutionary Genetics Analysis Version 7.0 for Bigger Datasets. Molecular Biology and Evolution.

