# Supplementary material for: Oral microbiome diversity in chimpanzees from Gombe National Park
Source: Sci Rep. 2019 Nov 22;9:17354. doi: 10.1038/s41598-019-53802-1 (PMC6874655; doi:10.1038/s41598-019-53802-1)
Supplement: Supplementary file 1 — Supplemental Figure 1 [file 41598_2019_53802_MOESM1_ESM.pdf]

Oral microbiome diversity in chimpanzees from Gombe National Park

Andrew T. Ozga[1,2,3]*, Ian Gilby[2,4], Rebecca S. Nockerts[5], Michael L. Wilson[5,6], Anne Pusey[7], and Anne C. Stone[1,2,4].

[1] Center for Evolution and Medicine, Arizona State University, Tempe, Arizona
[2] Institute of Human Origins, Arizona State University, Tempe, Arizona
[3] Halmos College of Natural Sciences and Oceanography, Nova Southeastern University, Fort Lauderdale, Florida
[4] School of Human Evolution and Social Change, Arizona State University, Tempe, Arizona
[5] Department of Anthropology, University of Minnesota, Minneapolis, Minnesota
[6] Department of Ecology, Evolution, and Behavior, University of Minnesota, Minneapolis, Minnesota
[7] Department of Evolutionary Anthropology, Duke University, Durham, North Carolina

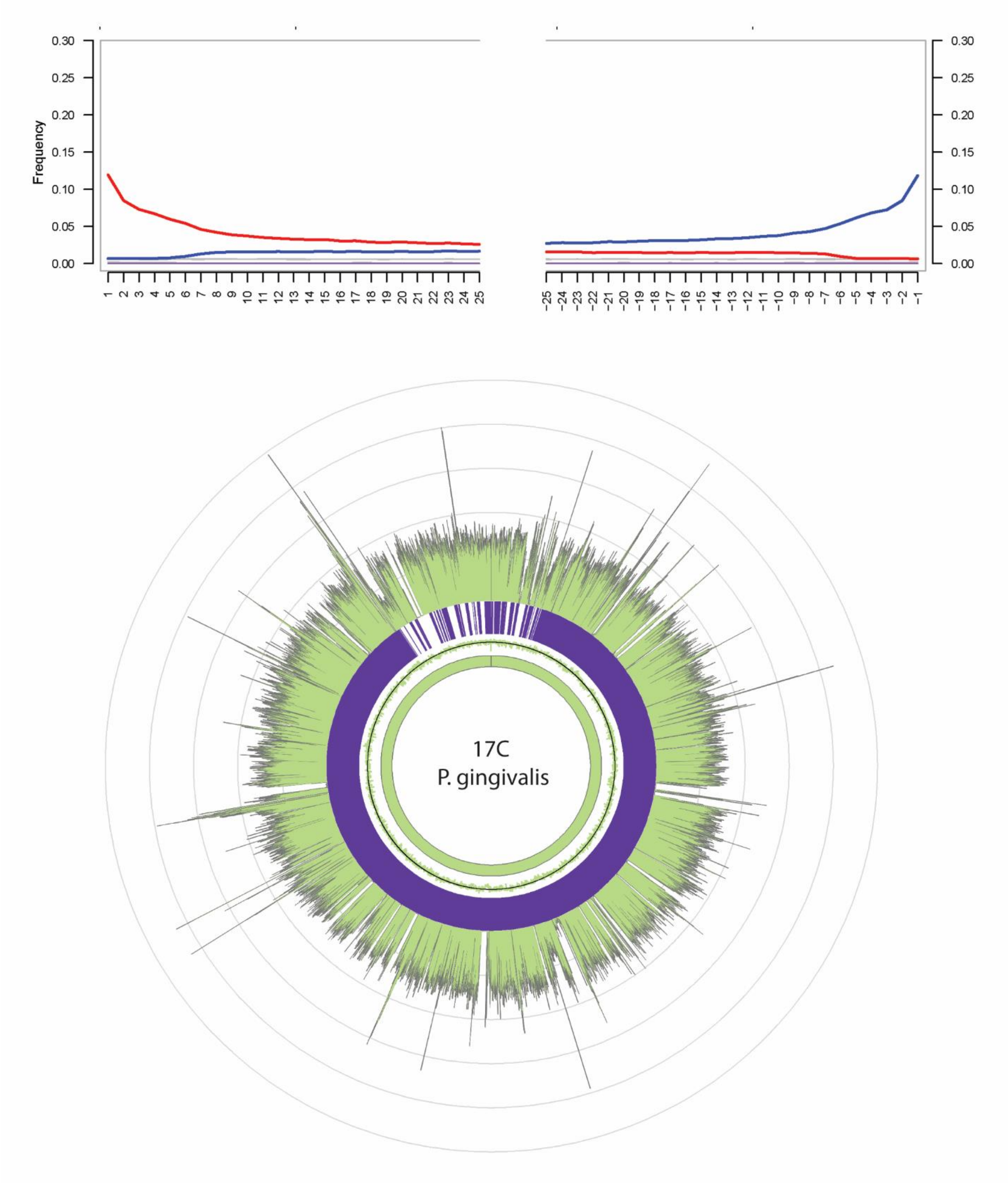

Supplementary Figure 1: A damage profile and Circos plot for *Porphyromonas gingivalis* from sample 17C. The 'smile' pattern in the damage profile is indicative of slightly damaged DNA. For the Circos plot, gray bars radiating from center indicate 25x to 125x coverage (in multiples of 25x). Total coverage is in green (250bp windows), while annotated genes are represented by the purple ring and GC coverage is represented by the inner green circle (250bp windows) with the gray line representing the average
